# Direct reprogramming of porcine fibroblasts to neural progenitor cells

**DOI:** 10.1007/s13238-013-0015-y

**Published:** 2014-02-04

**Authors:** Xiu-Ling Xu, Ji-Ping Yang, Li-Na Fu, Ruo-Tong Ren, Fei Yi, Keiichiro Suzuki, Kai Liu, Zhi-Chao Ding, Jing Qu, Wei-Qi Zhang, Ying Li, Ting-Ting Yuan, Guo-Hong Yuan, Li-Na Sui, Di Guan, Shun-Lei Duan, Hui-Ze Pan, Ping Wang, Xi-Ping Zhu, Nuria Montserrat, Ming Li, Rui-Jun Bai, Lin Liu, Juan Carlos Izpisua Belmonte, Guang-Hui Liu

**Affiliations:** 1 National Laboratory of Biomacromolecules, Institute of Biophysics, Chinese Academy of Sciences, Beijing, 100101 China; 2 Department of Molecular and Cellular Physiology, Stanford University School of Medicine, Stanford, CA 94305 USA; 3 Gene Expression Laboratory, Salk Institute for Biological Studies, 10010 North Torrey Pines Road, La Jolla, CA 92037 USA; 4 State Key Laboratory of Medicinal Chemical Biology, College of Life Sciences, Nankai University, Tianjin, 300071 China; 5 Center for Regenerative Medicine in Barcelona, Dr. Aiguader 88, 08003 Barcelona, Spain; 6 Beijing Institute for Brain Disorders, Beijing, 100069 China

**Dear Editor,**

Neural progenitor cells (NPCs) have proven potential to facilitate mechanistic studies of neurological disorders *in vitro*, as well as the discovery of new medicines. In addition, NPCs have been proposed as promising cell sources for cell replacement therapy of neurological diseases (Liu et al., 2012b). For these areas of study, experimental animals are indispensable models. Among the possible animal species, pigs are advantageous compared to rodents because of their physiological and anatomical similarities to humans (Lind et al., 2007). Despite the shown advantages of porcine models in different fields, their applications are significantly restricted due to the limited access of porcine cells, including NPCs. To date, encouraging breakthroughs have been made in obtaining NPCs from a series of species by different methods, including primary cell isolation from tissues, differentiation from pluripotent stem cells, and direct reprogramming from other somatic cells (Vierbuchen et al., 2010; Giorgetti et al., 2012; Lujan et al., 2012; Thier et al., 2012; Zhang et al., 2013).

Here, we report the successful generation of induced porcine NPCs (ipNPCs) from porcine fetal fibroblasts (PFFs) (Fig. S1A, upper panel). Using our method, functional ipNPCs can be readily obtained via direct cell reprogramming without going through a pluripotent state. We show that ipNPCs retain the ability for long-term culture and efficient neural differentiation *in vitro*. Moreover, ipNPCs could effectively integrate into the local neural network after cell transplantation *in vivo*.

In order to initiate the direct cell reprogramming, we sought to prime PFFs using non-integrative episomal vectors expressing reprogramming factors (Oct4, Sox2, Klf4, Lin28, and L-Myc) (Li et al., 2011) and then subjected the cells to human embryonic stem cell-amenable culture conditions (Liu et al., 2011). Three weeks later, ~ 10 colonies emerged from 5 × 10^5^ transduced PFFs plated on mouse embryonic fibroblasts (MEFs). These colonies did not exhibit characteristic morphology of porcine induced pluripotent stem cells (iPSCs) including high nucleus to cytoplasm ratio and clear colony boundaries, as reported in porcine iPSCs generated by viral vector-mediated methods (Ezashi et al., 2009; Wu et al., 2009). Instead, they exhibited irregular and unclear boundaries with filament-like cells spreading out (Fig. S1A, bottom panel). When a treatment of 50 μmol/L sodium butyrate (an inhibitor of histone deacetylases) was applied during the reprogramming, a slightly higher number of colonies with similar morphology emerged (data not shown). Immunofluorescence staining showed that these colonies expressed Sox2 at low levels and were negative for pluripotency markers Oct4 and Nanog (Fig. S1B). To initiate the neural commitment, these colonies were mechanically picked and seeded to MEFs supplied with neural stem cell culture medium (NSM) (Liu et al., 2012a). After a 10-day induction in NSM, the formation of neural rosettes was observed (Fig. 1A), which resembles an early stage of neurodevelopment. Immunofluorescence staining of neural rosettes confirmed the presence of an ipNPC population by expression of both Sox2 and Nestin (Fig. 1A). These neural rosette ipNPCs were then individualized, and subcultured in NSM on Matrigel. Under this condition, ipNPCs expanded in monolayer and over 95% of them were positive for Pax6 (Fig. 1B). Quantitative real-time PCR (qPCR) analysis further demonstrated the induction of NPC markers (NCAM, Nestin, and Pax6) in ipNPCs compared to their parental PFFs (Fig. 1C). We further determined the possible presence of residual or integrated episomal vectors in ipNPCs by genomic qPCR analysis of EBNA-1, a viral element of episomal vectors originating from Epstein-Barr virus (Li et al., 2011). The results showed that ipNPCs and PFFs contained almost undetectable levels of EBNA-1 (~ 0.0001–0.001 copies per cell), while episomal vector-transfected PFFs showed nearly 100 copies per cell, implying the absence of EBNA-1 in the ipNPC genome (Fig. S1C). The negative readout from this analysis relieved safety concerns to use ipNPCs for cell transplantation in the future. Also, ipNPCs had been robustly maintained for over 12 passages without significant signs of losing potency, suggesting an ability of long term self-renewal of ipNPCs *in vitro*. Meanwhile, when cultured on low-attachment plates, ipNPCs formed neurospheres spontaneously (Fig. S1D), further demonstrating neural stem cell identity and robust viability.

**Figure 1:**
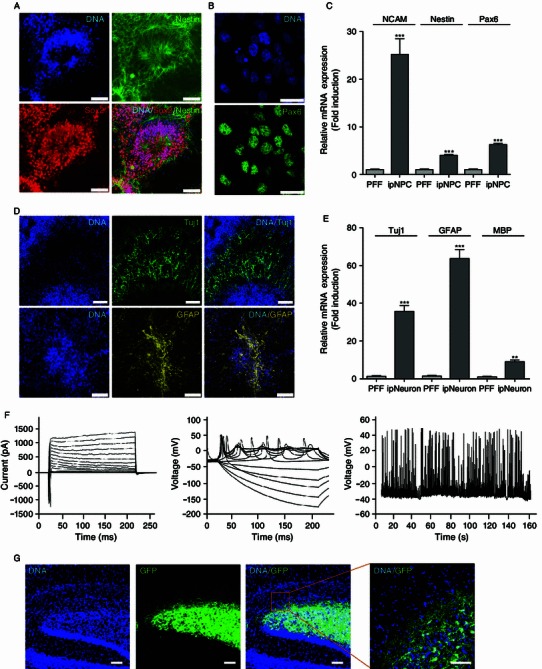
**Direct reprogramming of porcine fetal fibroblasts (PFFs) to induced porcine neural progenitor cells (ipNPCs)**. (A) Immunofluorescence staining of ipNPCs derived on MEFs showing rosette morphology and expressions of neural progenitor cell markers Nestin (in green) and Sox2 (in red). Scale bars, 75 μm. (B) Immunofluorescence staining of Pax6 positive ipNPCs cultured on matrigel. Scale bars, 25 μm. (C) Gene expression analysis showed that the neural progenitor cell markers (NCAM, Nestin, Pax6) were induced in the direct conversion of PFFs to ipNPCs. All values are relative to PFFs and shown as mean ± s.e.m. (*n* = 3). ***P < 0.001. (D) Differentiation of ipNPCs into neurons (Tuj1, Green) and glial cells (GFAP, Yellow) *in vitro*. Scale bars, 100 μm. (E) Quantitative PCR analysis showed the induction of neuronal specific marker (Tuj1), glial specific marker (GFAP), and oligodendrocyte marker (MBP) after spontaneous differentiation of ipNPCs. All values are relative to PFFs and shown as mean ± s.e.m. (*n* = 3). **P < 0.01, ***P < 0.001. (F) Representative traces of electrophysiology recording of differentiated neurons at day 30. Inward fast inactivating sodium currents and outward currents were observed in differentiated neurons by whole cell current recording (left). Action potentials (APs) were elicited by step-current injections (middle). Spontaneous APs firing from ipNeuron were recorded (right). (G) *In vivo* transplantation of ipNPCs. Overview of transplanted ipNPCs (GFP + , Green) cells in the dentate gyrus (DG) of brain from NOD/SCID mice at 4 weeks after ipNPCs transplantation. Blue, DNA; Green, GFP

Next, in order to assess the neural differentiation potency of ipNPCs, *in vitro* spontaneous neural differentiation was performed. After three weeks of culturing in spontaneous neural differentiation medium (NDM), the cell bodies of most cells were clustered and long neurites protruded. Immunofluorescence staining showed that the majority of differentiated cells were Tuj1 positive neurons while GFAP positive glial cells were also present (Fig. 1D). Correspondingly, the mRNA levels of Tuj1 and GFAP significantly increased after ipNPCs spontaneous differentiation (Fig. 1E). In addition, up-regulation of myelin basic protein (MBP), which is enriched in oligodendrocytes, was also observed by qPCR (Fig. 1E), suggesting a possible existence of oligodendrocytes in the differentiated derivatives. Taken together, our data indicated multipotent neural differentiation potential of ipNPCs.

Subsequently, in order to assess the functional membrane properties of ipNPC-derived neurons (ipNeurons), electrophysiology recordings were performed after thirty days of spontaneous differentiation. Whole-cell patch-clamp recordings on ipNeurons revealed voltage-dependent currents including rapidly inactivating inward currents and persistent outward currents in response to depolarization voltage steps, which reflected open and closed states of sodium channels and potassium channels respectively (Fig. 1F, left). Current clamp recordings demonstrated that the ipNeurons could generate action potentials, an evident membrane characteristic of excitable cells (Fig. 1F, middle). Meanwhile, spontaneous action potentials were also observed in differentiated ipNeurons (Fig. 1F, right). Collectively, these data demonstrated that ipNPCs are capable of differentiating into excitable neurons *in vitro*.

Finally, we explored the neural differentiation potency of ipNPCs *in vivo*. The ipNPCs were labeled with GFP by lentiviral vectors, and then transplanted into the dentate gyrus (DG) of NOD/SCID mice. Four weeks after transplantation, brains of recipient mice were sectioned and analyzed. We found that most GFP-labeled cells were localized in the DG region, indicating a robust survival of ipNPCs *in vivo* (Fig. 1G). We further observed GFP positive neurons with complex branching morphology that were present at neighboring zones of the DG region (Fig. 1G), which suggested that ipNPCs were able to effectively integrate into the local neural network after transplantation. No teratoma formation was observed in any mouse brains examined, which further supports the safety of ipNPCs *in vivo*. (Data not shown)

In summary, we report here a new strategy to obtain integration-free functional porcine neural progenitor cells by direct reprogramming of porcine fetal fibroblasts *in vitro*. For the first time, porcine neural progenitor cells were directly generated from somatic cells, and functionally characterized both *in vitro* and *in vivo*. Considering the importance of pigs as a model species, a sufficient supply of functional porcine neural progenitor cells are of great interest in translational medicine studies of neuroscience. However, the difficulties to establish porcine pluripotent stem cells including embryonic stem cells and integration-free iPSCs limit the production of porcine NPCs through traditional cell differentiation approaches (Wu et al., 2009; Rasmussen et al., 2011; Liu et al., 2012c; Fan et al., 2013). Therefore, how to obtain porcine NPCs directly from the somatic cells is attracting a lot of attention in the field. Similar to many other direct reprogramming methods, our strategy bypassed obstacles in establishing porcine pluripotent stem cells. Moreover, our method provided a robust and efficient way of generating porcine NPCs with low risk of tumor formation. To our knowledge, this is the first attempt to direct reprogram somatic cells into neural progenitor cells using the porcine species. As a promising species of model animals, the ipNPCs generated in our study may provide an exciting tool to bridge the present gaps in neuroscience studies between rodents and humans.

## Electronic supplementary material

Below is the link to the electronic supplementary material.

Supplementary material 1 (PDF 43 kb)

Supplementary material 2 (PDF 10 kb)

Supplementary material 3 (PDF 135 kb)
