# Supplementary material for: Direct reprogramming of porcine fibroblasts to neural progenitor cells
Source: Protein Cell. 2014 Feb 4;5(1):4–7. doi: 10.1007/s13238-013-0015-y (PMC3938843; doi:10.1007/s13238-013-0015-y)
Supplement: Supplementary file 1 — Supplementary material 1 (PDF 43 kb) [file 13238_2013_15_MOESM1_ESM.pdf]

Supplemental Material and Methods

Materials and Methods

Primary porcine fibroblast Cell culture

Porcine fetal fibroblasts (PFFs) were cultured as previously described (Liu et al., 2012) in PFF medium: high glucose Dulbecco's modified Eagle's medium (DMEM; Invitrogen), 10% fetal bovine serum (FBS; Invitrogen), 1×GlutaMAX (Invitrogen), 1×penicillin–streptomycin (Invitrogen).

Direct reprogramming and neural differentiation

Two million PFFs were electroporated with 1.5 μg of each episomal vectors: pCXLE-hOct3/4 (Addgene number 27076), pCXLE-hSK (Addgene number 27078), pCXLE-hUL (Addgene number 27080) and pCXLE-EGFP (Addgene number 27082)， and cultured on gelatin-coated plate. 4 days after the electroporation, cells were trypsinized and 5×10^5 of them were plated on MEF feeders in PFF medium. 24 hours after seeding, the medium were switched to human embryonic stem cell (hESC) medium: DMEM/F12 (Invitrogen), KO serum replacement (20%; Invitrogen), GlutaMAX (nvitrogen), penicillin–streptomycin (Invitrogen), non-essential amino acid solution (100μM; Invitrogen), 2-mercaptoethanol (100μM; Invitrogen), recombinant human fibroblast growth factor (10ng/ml; PeproTech). Medium was changed every 2 days during the reprogramming.

When colonies appeared at around 3 weeks, the clustered cells were picked mechanically and transferred onto MEF feeders containing neural stem cell medium (NSM): 50% DMEM/F12 (50%; Invitrogen), Neurobasal medium (50%; Invitrogen), 1×N2 supplement (Invitrogen), 1×B27 supplement (Invitrogen), 1×GlutaMAX (Invitrogen),

hLIF (10 ng/mL; Millipore), CHIR99021 (4μM;Tocris), SB431542 (3μM; Tocris) and Dorsomorphin(2μM; Tocris).

After NPC induction, the ipNPCs were individualized with Accumax (Millipore) and seeded on the matrigel-coated plates with NSM for further passaging.

For spontaneous differentiation of ipNPCs, 2×10^4 ipNPCs were dissociated by using Accumax (Millipore) and were then plated on matrigel-coated 24-well plates. Cells were cultured in NSM with medium change daily for first 3 days. Spontaneous differentiation was performed with medium change weekly for 4 weeks.

Quantitative real-time PCR

RNA were extracted from single cultures, using Trizol isolation method (Invitrogen) according to manufacturer's instructions. Two micrograms of RNA were reverse transcribed using the GoScript Reverse Transcription System (Promega). 20 nanogram of the reverse transcribed cDNA was used as the template for quantitative PCR reaction. Primers are listed in supplementary Table1.

Immunofluorescence staining

For immunofluorescence analysis, cells were fixed in 4% paraformaldehyde for 20 min at room temperature, followed by permeabilization in 0.1% TRITON-X100/PBS for 20 min. Blocking was performed with 10% Donkey Serum (Jackson Immunoresearch Laboratory) in PBS for 1 hrs. Samples were incubated with primary antibodies diluted in 1% blocking solution at 4°C overnight, washed twice in PBS and incubated with secondary antibody diluted in 1% blocking solution for 45 mins at room temperature, counterstained with

HOECHST and mounted with VECTASHIELD mounting solution (Vector Laboratories). The following antibodies were used for our analysis: anti-Sox2 (R&D; 1:250), anti-Nestin (Millipore, MAB5326, 1:500) anti-Pax6 (Abcam, 1:200), anti-Tuj1 (Sigma, 1:200), anti-GFAP (DAKO, 1:200). Images were collected using a Leica SP5 confocal microscope.

Integration detection of episomal vectors

The cell pellets were lysed in lysis buffer (100mM Tris-HCl PH 8.5, 5mM EDTA, 0.2% SDS, 200mM NaCl) with proteinase K at 50°C for 1hr. DNA was extracted with phenol/chloroform/octanol (25:24:1) and chloroform/octanol (24:1) sequentially. The isolated DNAs were precipitated in cold ethanol and used for genomic quantitative PCR analysis. The genomic qPCR was performed as described previously with minimal optimization of primers (Okita et al., 2011). Primers are listed in supplementary Table1.

Electrophysiology recording

Recordings were performed on differentiated neurons at day 30 after spontaneous differentiation. The neurons with well-developed neurites were chosen for recording. Cells were perfused with the following composition: 140mM NaCl, 5mM KCl, 2mM $CaCl_2$, 2mM $MgCl_2$, 15mM HEPES and 25mM glucose, pH adjusted to 7.4. Pipette solution contained: 130mM K-gluconate, 10mM KCl, 0.5 mM $CaCl_2$, 15 mM HEPES, 5mM EGTA, 8mM NaCl, 2mM MgATP, 0.3mM Na_2GTP and 10mM glucose, pH adjusted to 7.2 with KOH. Action potentials (APs) were recorded with current-clamp whole-cell configuration. Membrane potentials were kept around −30 to −40mV, and step

currents were injected to elicit action potentials. Whole-cell currents including sodium currents, potassium currents were recorded at a holding potential of −40mV, voltage steps ranging from −50mV to +60mV were delivered at 10mV increments.

Transplantation of ipNPCs

ipNPCs were labeled with GFP by lentiviral infection and 5×10^5 cells resuspended in 145mM Glucose/0.9% Saline were transplanted into dentate gyrus (DG) of NOD/SCID mice. Mice were sacrificed 4 weeks after transplantation. The brains were dissected out and fixed in 4% paraformaldehyde overnight followed by dehydration in 0.1M PBS containing 30% sucrose for 2 days at 4°C. Consecutive coronal sections were sliced using a Leica SM 2000R Sliding Microtome. Sections with GFP positive cells were picked for the following immunofluorescence staining.

.

Statistical analysis.

All experiments were performed in triplicate at least twice. Data are presented as mean±s.e.m. Statistical significance of differences between groups was determined using unpaired Student's t-test.

Liu, K., Ji, G., Mao, J., Liu, M., Wang, L., Chen, C., and Liu, L. (2012). Generation of porcine-induced pluripotent stem cells by using OCT4 and KLF4 porcine factors. Cell Reprogram 14, 505-513.

Okita, K., Matsumura, Y., Sato, Y., Okada, A., Morizane, A., Okamoto, S., Hong, H., Nakagawa, M., Tanabe, K., Tezuka, K., *et al.* (2011). A more efficient method to generate integration-free human iPS cells. Nat Methods 8, 409-412.
