# Supplementary material for: Direct reprogramming of porcine fibroblasts to neural progenitor cells
Source: Protein Cell. 2014 Feb 4;5(1):4–7. doi: 10.1007/s13238-013-0015-y (PMC3938843; doi:10.1007/s13238-013-0015-y)
Supplement: Supplementary file 2 — Supplementary material 2 (PDF 10 kb) [file 13238_2013_15_MOESM2_ESM.pdf]

Supplementary Table1
List of qPCR primers used in this study

pig-Pax6 F	GAGTTCTTCGCAACCTGGCTA
pig-Pax6 R	TGGTATTCTCTCCCCCTCCTT
pig-Tuj1 F	GTGGTGCGGAAGGAGTGTG
pig-Tuj1 R	TGGTGGATGGACAGCGTGG
pig-NCAM F	CGGAGGGAAGCACACGGAG
pig-NCAM R	CGCTTTGCTCTCGTTCTCCTT
pig-GFAP F	TTGACCTGCGACGGGAGTC
pig-GFAP R	AGGTGGCGATCTCGATGTCC
pig-GAPDH F	TCGGAGTGAACGGATTTG
pig-GAPDH R	CCTGGAAGATGGTGATGG
pig-MBP F	GAGGCAGAGCTCCTGACTACAAA
pig-MBP R	GTCCCGTCCTCCCAGCTT
pig-Nestin F	GTCCGCTGCTGCTCCCTTGG
pig-Nestin R	AGGGGCGCTTGGGGACATCT
EBNA-1 F	ATCAGGGCCAAGACATAGAGATG
EBNA-1 R	GCCAATGCAACTTGGACGTT
