# Supplementary material for: Direct reprogramming of porcine fibroblasts to neural progenitor cells
Source: Protein Cell. 2014 Feb 4;5(1):4–7. doi: 10.1007/s13238-013-0015-y (PMC3938843; doi:10.1007/s13238-013-0015-y)
Supplement: Supplementary file 3 — Supplementary material 3 (PDF 135 kb) [file 13238_2013_15_MOESM3_ESM.pdf]

SUPPLEMENTARY FIGURE LEGENDS.

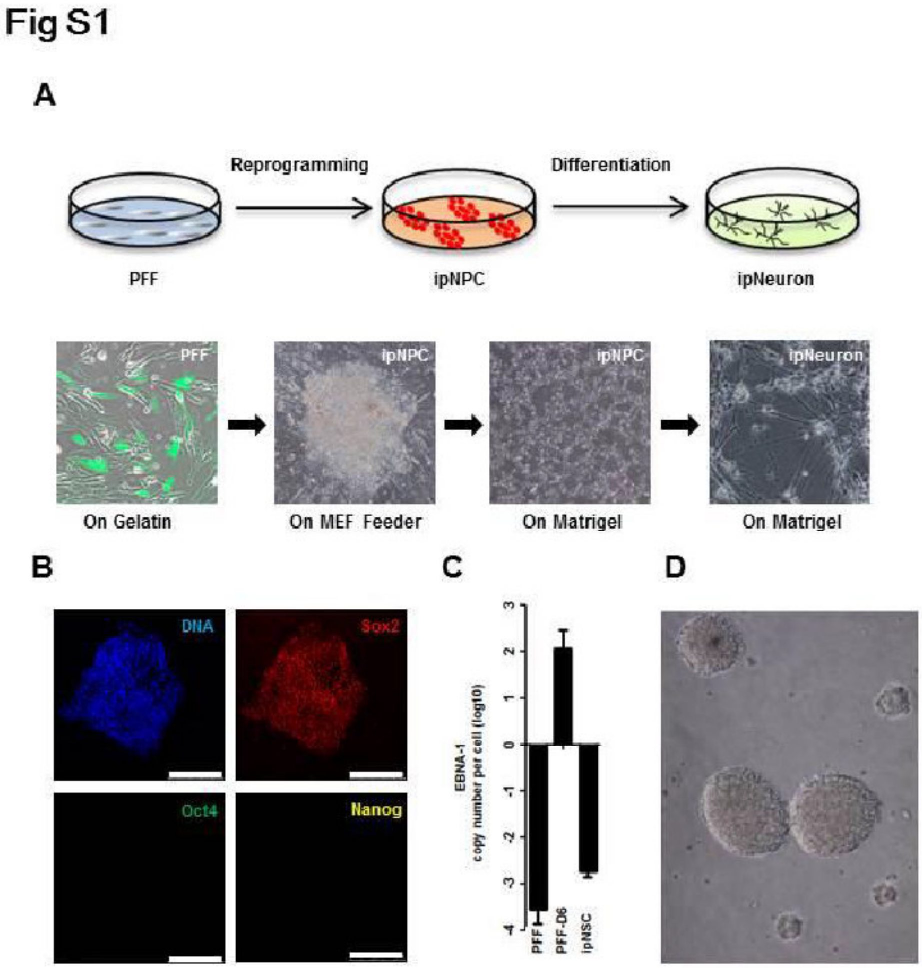

Fig S1. (A) A schematic view of direct conversion of porcine fetal fibroblasts to neural progenitor cells. Lower panel showed bright-field images of the direct reprogramming of porcine fetal fibroblasts into neural cells. Porcine fetal fibroblasts were electroporated with episomal factors and cultured on gelatin-coated plate. The successfully transduced PFFs were GFP positive and re-seeded on mouse embryonic fibroblast (MEF) feeders. Reprogramming and expanding on matrigel-coated plate in neural stem cell culture medium enabled the direct cell conversion into induced neural progenitor cells. Also, the generated ipNPCs were capable of differentiating into neural cells. (B) Immunofluorescence staining showed that the induced colonies on MEF feeders were negative for Oct4 and Nanog, but positive for Sox2. (C) Copy numbers of EBNA-1 of episomal vectors in ipNPCs. PFFs electroporated 6 days (PFF-D6) were analyzed as positive control, while PFFs as negative control. (D) The ipNPCs formed neurospheres in a suspension culture.
